# Application of extracellular flux analysis for determining mitochondrial function in mammalian oocytes and early embryos

**DOI:** 10.1038/s41598-019-53066-9

**Published:** 2019-11-14

**Authors:** Bethany Muller, Niamh Lewis, Tope Adeniyi, Henry J. Leese, Daniel R. Brison, Roger G. Sturmey

**Affiliations:** 1 Centre for Atherothrombosis and Metabolic Disease, Hull York Medical School, University of Hull, Hull, HU6 7RX UK; 2 Institute of Aging and Chronic Disease, University of Liverpool, Liverpool, UK; 3 Department of Reproductive Medicine, Manchester University NHS Foundation Trust, Manchester Academic Health Sciences Centre UK, Manchester, UK; 4 Maternal and Fetal Health Research Centre, School of Medical Sciences, University of Manchester UK, Manchester, UK

**Keywords:** Embryology, Biological techniques

## Abstract

Mitochondria provide the major source of ATP for mammalian oocyte maturation and early embryo development. Oxygen Consumption Rate (OCR) is an established measure of mitochondrial function. OCR by mammalian oocytes and embryos has generally been restricted to overall uptake and detailed understanding of the components of OCR dedicated to specific molecular events remains lacking. Here, extracellular flux analysis (EFA) was applied to small groups of bovine, equine, mouse and human oocytes and bovine early embryos to measure OCR and its components. Using EFA, we report the changes in mitochondrial activity during the processes of oocyte maturation, fertilisation, and pre-implantation development to blastocyst stage in response to physiological demands in mammalian embryos. Crucially, we describe the real time partitioning of overall OCR to spare capacity, proton leak, non-mitochondrial and coupled respiration – showing that while activity changes over the course of development in response to physiological demand, the overall efficiency is unchanged. EFA is shown to be able to measure mitochondrial function in small groups of mammalian oocytes and embryos in a manner which is robust, rapid and easy to use. EFA is non-invasive and allows real-time determination of the impact of compounds on OCR, facilitating an assessment of the components of mitochondrial activity. This provides proof-of-concept for EFA as an accessible system with which to study mammalian oocyte and embryo metabolism.

## Introduction

The re-emergence of the importance of metabolism in health and disease has stimulated investigations of mitochondrial function in the earliest stages of mammalian development. Mitochondria are abundant in the mammalian egg^1,2^, with mitochondrial DNA (mtDNA) copy number rising during oocyte maturation^3^ to a level at least ten-fold greater than somatic cells^4^. By contrast, in the early embryo, mitochondria do not replicate until the blastocyst stage or later^5,6^; thus, the pool of oocyte mitochondria at the point of ovulation support the entire pre-implantation period of development. In the oocyte and cleavage stage embryo, mitochondria exist in an immature though functional form^7,8^. Acquisition of a typical mitochondrial morphology does not occur until blastocyst formation, corresponding to a period of increased energy demand^9–13^.

Mitochondrial oxidative phosphorylation, measured as the oxygen consumption rate (OCR)^14^, is the largest contributor to cellular ATP demand during preimplantation development^12,15,16^. A number of techniques have been used to measure OCR in oocytes and embryos, including the Cartesian diver^17^, microspectrophotometry^18^, pyrene ultramicrofluorescence^10,11^, scanning electron microscopy^19,20^ and micro-respirometry^21,22^. Combined, these approaches have been instrumental in defining the overall metabolism of oocytes and early embryos, and have yielded remarkably consistent data. Importantly, OCR of mammalian embryos has been reported to correlate with reproductive physiological outcomes including oocyte maturation^23^, embryo morphology^13,19,20^, implantation potential^24^, and pregnancy rate^13^. Generally, ‘mid-range’ or ‘Goldilocks-range’ OCR values are associated with higher oocyte/embryo quality and viability^25^.

Despite research into embryo metabolism, a more comprehensive picture of OCR in oocytes and embryos in terms of the components of mitochondrial oxygen flux has remained elusive. A major limiting factor has been the lack of appropriate technology since current methods are time-consuming and technically demanding. This has restricted their applicability to Assisted Reproductive Technology (ART) clinics where they might potentially be used to support selection of healthy embryos or as a tool for high-throughput screening or research to facilitate understanding of this critical element of embryo function. Recently, the emergence of extracellular flux analysis (EFA) technologies developed by Seahorse Bioscience (Agilent Technologies) has had a significant impact on metabolic research in a range of systems^26^. EFA permits the real-time measurement of metabolism and the systematic evaluation of components of cellular oxygen consumption. We have applied this method for the measurement of OCR by oocytes from a range of mammalian species, including the human. We have then explored the components of OCR on bovine embryos using EFA and discovered that mitochondrial function changes post fertilisation and between the cleavage and blastocyst stages.

## Results

### Establishment of extracellular flux analysis of bovine COCs

Initially, bovine oocytes at the germinal vesicle (GV) stage contained within intact cumulus complexes (COCs) were used to validate a method for the reproducible measurement of OCR. A linear relationship (r^2^ = 0.90; p = 0.0042) was observed between the number of COCs and OCR, when comparing groups of between 1 and 25 COCs per well (Supplementary Fig. S1). A comparison of group size (3 or 6) demonstrated that groups of 3 COCs were around the limit of detection of the assay system. Subsequent studies were therefore performed on groups of 6 COCs where possible.

### Oxygen consumption in mammalian oocytes – the contribution of cumulus and the impact of *in vitro* maturation and *in vitro* fertilisation

**Figure 1:**
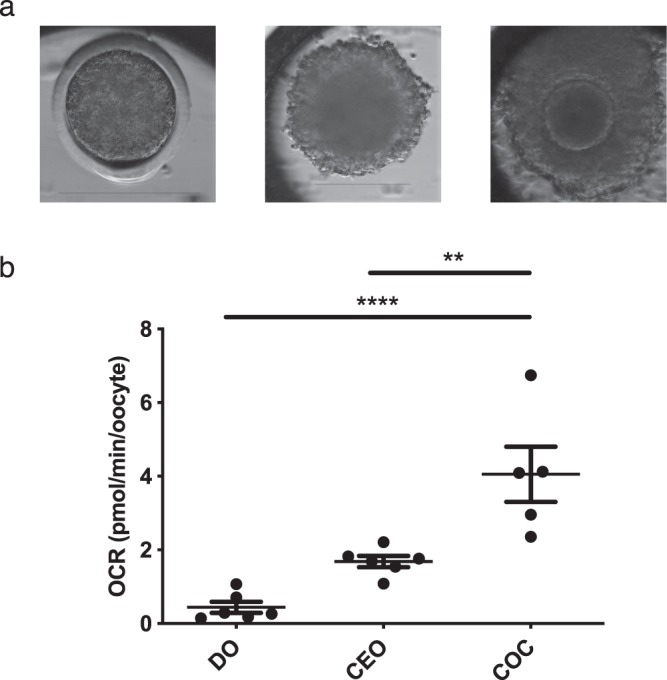
Contribution of the cumulus to bovine oocyte oxygen consumption at the GV-stage. (**a**) Time-lapse images of a fully denuded oocyte (DO), corona-enclosed oocyte (CEO) and intact cumulus oocyte complex (COC). Scale bars depict 150 µm. (**b**) Basal OCR of DO, CEO and COCs. Data presented as mean ± SEM, representing data from 6 wells (36 oocytes) per group. **Indicates p < 0.01, ****indicates p < 0.001. Consumption of oxygen was significantly greater than 0 (p < 0.05) confirmed by Wilcoxon Signed Rank Test.

The contribution of cumulus cells to GV-stage oocyte respiratory activity was investigated by comparing the OCR of fully denuded oocytes (DO), corona-enclosed oocytes (CEOs) containing only the innermost 2–3 layers of cumulus cells, and fully intact COCs (Fig. 1). OCR was significantly lower in DOs (0.44 ± 0.15 pmol/min/oocyte) and CEOs (1.68 ± 0.15 pmol/min/oocyte) compared to intact COCs (4.05 ± 0.75 pmol/min/oocyte). CEOs were selected for use in subsequent studies, to more closely resemble the physiological state of oocytes *in vivo* through maintaining the interaction between the two cell types, while keeping biological variation to a minimum.

**Figure 2:**
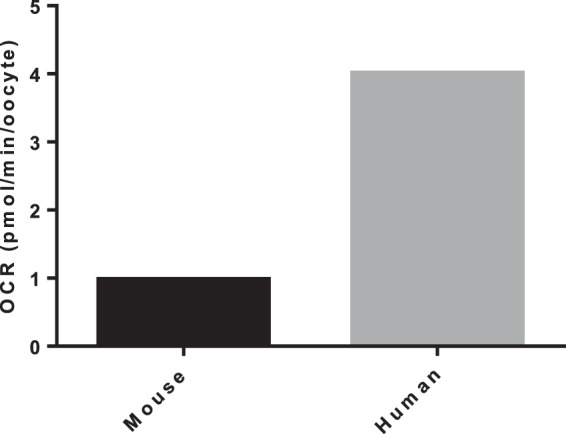
Basal OCR in denuded M-II mouse and human oocytes. Basal OCR mouse (8 oocytes) and human (6 oocytes) are indicated. Data is from a single replicate.

EFA was then applied to small groups of mouse and human denuded M-II oocytes in order to demonstrate the applicability of the system across species (Fig. 2). However, due to limited availability of material and the sensitivity limits of the system, the data represents a single well of oocytes thus demonstrating proof of principle but not allowing quantitative comparison.

**Figure 3:**
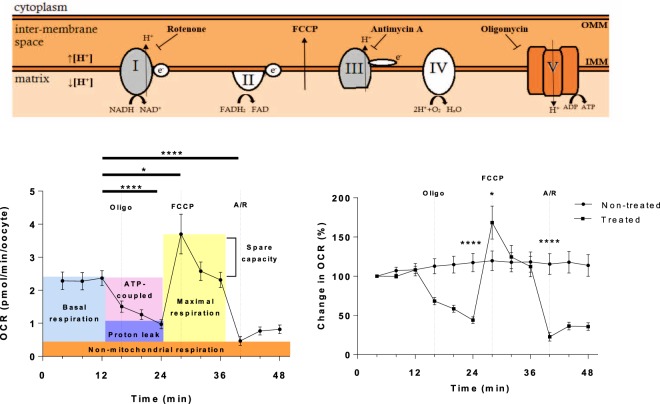
Application of mitochondrial inhibitors oligomycin, FCCP and antimycin A/rotenone to GV-stage oocytes. (**a**) Indicates the drug targets of Oligomycin, FCCP, Antimycin A and Rotenone. Oligomycin blocks ATP-synthase, FCCP dissipates the proton gradient between the matrix and inner membrane space, and antimycin A and rotenone inhibit complexes III and I respectively. (**b**,**c**) The response of GV-stage CEOs to the sequential addition of 1 μM oligomycin, 5 μM FCC and 2.5 μM A/R, depicted as (**b**) OCR (pmol/min/oocyte) or (**c**) as compared to control non-treated oocytes, shown as percentage of the third basal measurement. (mean ± SEM, representative of 12 wells (72 CEOs) treated and 13 wells (78 CEOS) untreated). *Represents p < 0.05 and ****represents p < 0.0001.

In order to measure the components of oxygen consumption of bovine CEOs, it was necessary to optimise the concentration of a series of mitochondrial inhibitors used to disrupt mitochondrial function (Supplementary Fig. S3). Oligomycin inhibits ATP-synthase and can be used to indicate the proportion of O_2_ consumption directly coupled to ATP generation. Carbonyl cyanide-4-(trifluoromethoxy)phenylhydrazone (FCCP) is a mitochondrial uncoupler which dissipates the proton gradient between the inter membrane space (IMS) and the matrix allowing the measurement of maximal OCR. Added together, Antimycin A, a complex III inhibitor, and rotenone, a complex I inhibitor, combine to inhibit the ETC entirely; thus the proportion of OCR that is insensitive to the combined addition of Antimycin A and Rotenone (A/R) is considered to be non-mitochondrial in origin. 1 µM oligomycin, 5 µM FCCP and 2.5 µM A/R gave the maximal response without killing the CEOs. The OCR fell to 43.9 ± 4.1% of basal OCR in response to inhibition of ATP synthase with oligomycin, rose significantly to 68.4 ± 21.0% above basal upon disruption of the proton gradient with FCCP, and fell to 22.7 ± 5.5% of basal OCR after the combined addition of A/R which totally blocks mitochondrial function (Fig. 3). The per-well difference between non-mitochondrial and coupled OCR, proton leak, was 21.8 ± 6.2% (Fig. 3c). These same concentrations were applied to mouse and human oocytes to mitochondrial inhibitors, and demonstrated that OCR was indicative of oxidative phosphorylation due to the fact that responses were observed to some extent despite lack of optimization of concentrations (Supplementary Fig. S2).

**Figure 4:**
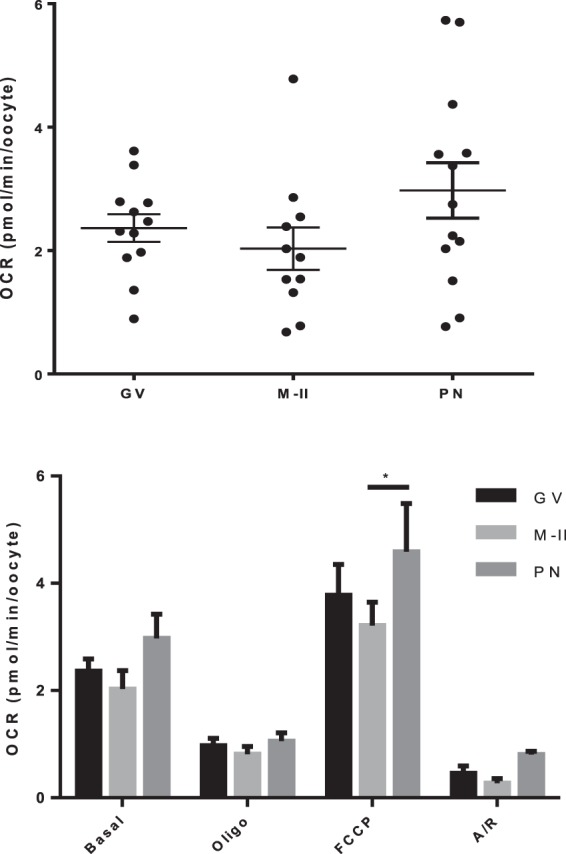
Oxygen consumption in bovine oocytes at GV, MII and PN stages. (**a**) Basal OCR of GV and MII-stage oocytes, and PN-stage zygotes (**b**) OCR of immature, mature and PN-stage zygotes in response to addition of 1 μM oligomycin, 5 μM FCCP and 2.5 μM A/R (representative of 12 wells (72 oocytes), 11 wells (66 oocytes) mature and 13 wells (78 zygotes) respectively). All data presented as mean ± SEM, representative of 12, 11 and 13 wells of oocytes (72 and 66) and zygotes (78) at each respective stage. *Depicts p < 0.05.

Having established the feasibility of measuring the components of OCR in CEOs, we next sought to discover whether the allocation of oxygen components changed during oocyte maturation and fertilisation. There was no difference in OCR (Fig. 4a) or respiratory chain constituents (Fig. 4b) between GV and MII oocytes. The optimal timing for pronuclear (PN) formation was determined as 9 hours post sperm addition (Supplementary Fig. S4). Presumptive zygotes at the PN stage with intact corona demonstrated a slight, non-significant increase in OCR to 2.97 ± 0.45 compared to mature oocytes (p = 0.17) and more notably a higher variability of OCR values. Further, maximal OCR of PN zygotes was significantly higher than in mature oocytes (Fig. 4b).

**Figure 5:**
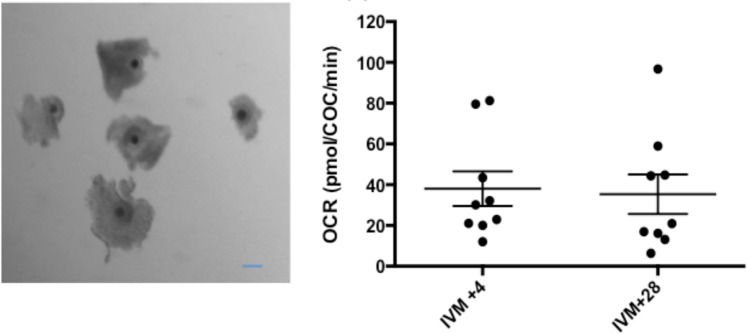
Basal OCR of equine cumulus enclosed oocytes at GV and MII stages of development. (**a**) Photomicrographs of equine COCs with compact cumulus. Scale bar depicts 100 µm (**b**) Basal OCR measured at 4 and 28 hours after the initiation of the 30 h *in vitro* maturation period (n = 9 wells, representative of 27 COCs). Oocytes were cultured in Seahorse XFp plates between measurements. All data are presented as mean ± SEM.

In order to examine the relationship in another species, 27 equine COCs were analysed across three replicates at the beginning and end of a 30 hour maturation period. Basal OCR did not vary (Fig. 5a) – with values of 38.1 ± 8.5 and 35.36 ± 9.7 pmol/COC/min after 4 and 28 hours respectively.

### Oxygen consumption in bovine embryos during preimplantation development *in vitro*

**Figure 6:**
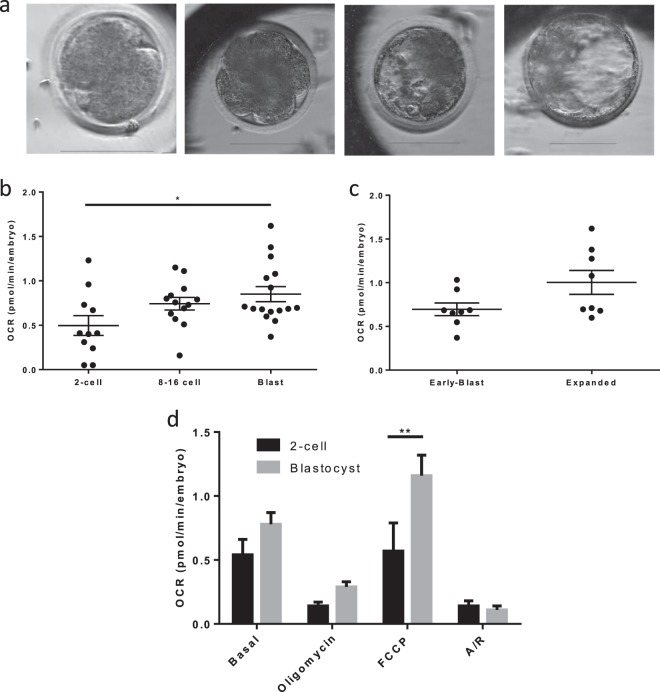
Oxygen consumption in embryos at cleavage and blastocyst stages. (**a**) Indicative time-lapse images of bovine embryos at 2-cell, 8–16 cell, full and expanded blastocyst stages. Scale bar depicts 100 µm. (**b**) Basal OCR in 2–4 cell, 8–16 cell and blastocyst stage embryos (representative of 66 2–4 cell embryos, 78 8–16 stage embryos, and 96 blastocysts). (**c**) OCR of early compared to expanded blastocysts (representative of 48 of each group). (**d**) OCR in response to inhibitors oligomycin, FCCP and A/R added sequentially at the 2-cell and blastocyst stage embryos (11 wells, 66 embryos per group). All data are presented as mean ± SEM. *Indicates p < 0.05, **p < 0.01.

We next tested whether the Seahorse anlyser could record OCR by pre-implantation embryos. In the bovine, there was a gradual increase in OCR as embryos progressed through the cleavage stage - with OCRs of 0.54 ± 0.12 and 0.74 ± 0.07 pmol/min at the 2–4 cell and 8–16 cell stages respectively, followed by a significant increase at the blastocyst stage to 0.85 ± 0.08 pmol/min/embryo (p = 0.02) (Fig. 6b). When the OCR was compared between early and expanded blastocysts, there was a small increase which approached significance (p = 0.066) (Fig. 6c). Importantly, exposing cleavage stage embryos to analysis of EFA did not impede development to blastocyst, an important developmental endpoint *in vitro* (Supplementary Fig. S5).

Having measured basal OCR, the components of respiration were analysed in embryos. In initial experiments, the concentrations of mitochondrial inhibitors oligomycin and antimycin/rotenone used for oocytes were found to be effective on embryos. By contrast, FCCP required further optimisation for use on embryos (Supplementary Fig. S6). Using the revised concentrations of mitochondrial inhibitors, maximal respiration was significantly higher in blastocyst stage embryos compared to early cleavage (p = 0.001); however OCR values in response to oligo and A/R indicative of coupled and non-mitochondrial respiration were unchanged between the stages (Fig. 6d).

**Figure 7:**
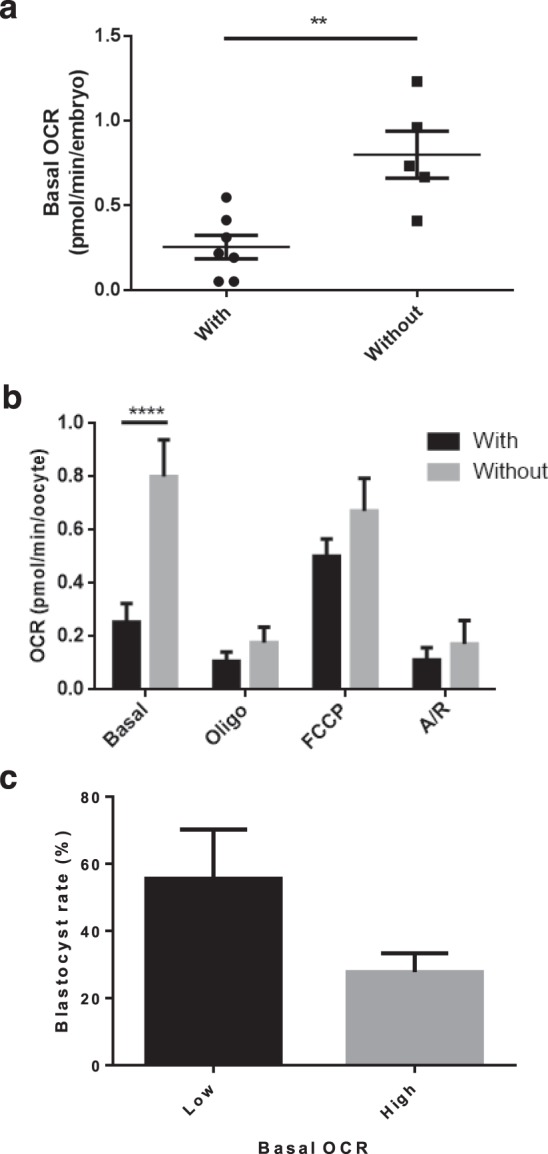
2–4 cell embryo mitochondrial activity. Early cleavage stage embryos showed variable response to FCCP. (**a**) Basal respiration (pmol/min/embryo) is shown for those with and without spare respiratory capacity (n = 7 and n = 5 respectively). (**b**) Drug response is shown across low and high groups. **Indicates p < 0.01, ****indicates p < 0.0001. (**c**) Blastocyst rate in embryos with low (ranging from 0.22–0.43 pmol/min/embryo) and high (ranging from 0.64–0.81 pmol/min/embryo) basal OCR at the cleavage stage (n = 1, representing 36 blastocysts).

Whist examining the components of OCR by 2 cell embryos, we observed a sub population that did not respond to FCCP, indicative of a lack of respiratory spare capacity. This was an unexpected finding which was explored in more detail (Fig. 7). This led to the finding that early cleavage stage bovine embryos that failed to respond to FCCP had a significantly elevated basal OCR compared to embryos that were able to respond to FCCP by increasing their overall OCR (Fig. 7a,b). 36 distinct 2–4 cell embryos were allocated into groups following basal EFA depending on whether they had ‘low’ (0.22–0.43 pmol/min) or ‘high’ (0.64–0.81 pmol/min) OCR, and cultured for a further 5 days to determine whether the distinct groups showed differing ability to reach the blastocyst stage. The resulting blastocyst rates for those with ‘low’ OCR were observed to be slightly higher than those with ‘high’ (p = 0.28) (Fig. 7c).

**Figure 8:**
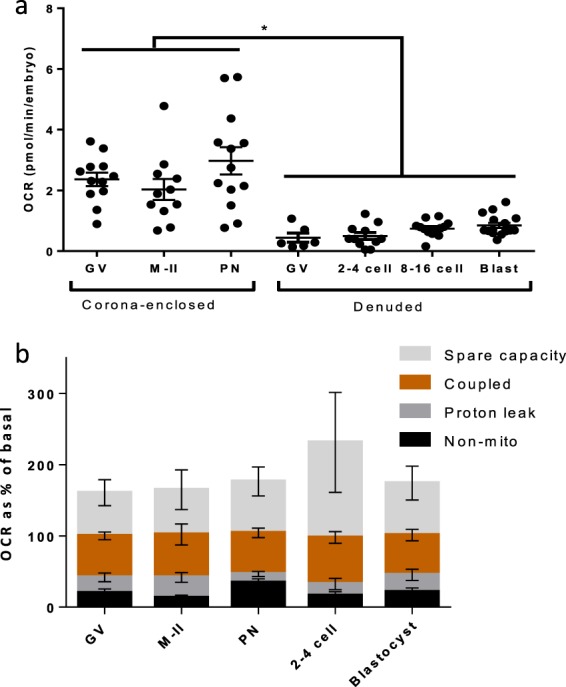
Summary of oxygen consumption across pre-implantation embryogenesis. (**a**) OCR of oocytes and embryos across pre-implantation development (representative of 72 GV-stage oocytes, 84 mII-stage oocytes, 78 PN-stage zygotes, 66 2–4 cell embryos, 78 8–16 stage embryos, and 96 blastocysts). (**b**) OCR in oocytes, PN-stage zygotes, 2–4 cell stage and blastocyst stage embryos in response to oligomycin, FCCP and A/R as indicative of mitochondrial parameters as a proportion of basal OCR (representative of 72 immature, 66 mature oocytes, 78 PN-stage zygotes and 66 embryos early cleavage and blastocyst stages). All data are presented as mean ± SEM. *Indicates p < 0.05.

Bovine embryos, at all stages consumed significantly less oxygen than corona-enclosed oocytes and zygotes in terms of basal (Fig. 8a) and maximal respiration (Fig. 8b). However, when the contributions of ATP-coupled, non-mitochondrial, proton leak and spare capacity to overall oxygen consumption across CEOs and embryos at key developmental stages were measured, there were no significant differences across development (Fig. 8c).

## Discussion

The metabolic processes supporting preimplantation development have been studied for more than 50 years^15,27^, with the aim of gaining fundamental understanding of the early mammalian embryo and identifying markers of embryo phenotype. While this research has provided a mature set of data describing embryo metabolism, the assays used are technically complex and require dedicated equipment sensitive enough to measure very small quantities of biological material. In particular, the measurement of OCR, which is a marker of global oxidative metabolism, has only been reported for oocytes and embryos by a handful of laboratories. Here, we describe the first use of EFA as an accessible system which can measure oxygen consumption and its components in small groups of mammalian oocytes and early embryos.

### Application of EFA to investigate the bovine cumulus-oocyte complex

The mammalian oocyte is supported by close association with granulosa-derived somatic cumulus cells. The metabolic co-operation between cumulus and oocyte is vital^28^, with cumulus cells being responsible for supplying key nutrients to the oocyte in the final phase of oocyte maturation^29–31^. After methods validation, the contribution of the cumulus cells to bovine oocyte respiration was determined. Cumulus cells are preferentially glycolytic^32^ and should therefore have only a minimal effect on oxygen consumption. However, we observed significantly lower OCR in denuded oocytes compared to COCs containing full cumulus contribution, indicating that the presence of cumulus cells does impact on the level of oxygen consumption in bovine oocytes. The data showing a significant difference between CEOs and COCs suggests that while each individual cumulus cell makes minimal contribution to OCR, the effect is amplified when the many cells work together, likely due to increased levels of signalling molecules as well as enhanced nutrient supply rather than the intrinsic OCR in the cumulus cells due to their comparatively low mitochondrial number and predominantly glycolytic nature. Overall, our data support the notion that cumulus cells have a significant impact on oocyte oxidative metabolism however further work is required to fully understand the nature of this observation.

### Application of extracellular flux analysis to mouse, human and equine oocytes

Using equine, mouse and human oocytes, the suitability of EFA to determine OCR in a variety of species was confirmed. The data were reassuringly close to those values obtained by previous methods; for example in the human, viable oocytes have been reported to consume 0.37 pmol/min^23^; in the mouse, 0.21 pmol/min^33^; and equine oocytes 3 pmol/min^22^; values close to our own. In the present work, mouse, human and equine oocytes showed higher basal respiration than bovine oocytes, however, the mouse and human oocytes used had failed to fertilise, suggesting they may not have been of optimal quality. Equine COC OCRs were approximately 10 times higher than bovine COCs. While the equine COCs were collected via follicular scraping rather than aspiration and likely had increased numbers of cumulus cells attached, the trend is in agreement with a previous equine study by Obeidat *et al*.^22^ in which denuded oocytes were used.

### Application of extracellular flux analysis to dissect mitochondrial function of bovine oocytes following maturation and fertilisation

The mitochondrial drugs oligomycin, FCCP and antimycin/rotenone were added systematically to dissect the components of OCR in mammalian oocytes. The data revealed that approximately 60% of OCR is coupled to ATP synthesis while a small but significant component of OCR (20%), is utilised for non-mitochondrial purposes. Bovine CEOs at the GV stage have significant respiratory spare capacity, consistent with previous observations in bovine oocytes^34^ and presumably required to satisfy dynamic energy demands as the oocyte undergoes maturation. These data confirm that the systematic interrogation of components of OCR in mammalian oocytes can be achieved through EFA.

Overall OCR did not change significantly over the course of culture in maturation media for bovine or equine oocytes. Contradictory results have been reported regarding the changes in OCR between the GV and MII stages^34^ which may reflect metabolic quiescence following meiotic progression and arrest at the M-II stage. However, in denuded pig and mouse oocytes, the opposite has been reported^12,33^ with M-II oocytes being more active than GV. This may reflect a species- specific difference, cumulus contribution, or be influenced by differences in IVM protocols. Moreover, it has previously been demonstrated that *in vitro* and *in vivo* matured oocytes show differential OCR profiles^34^.

In the corona-enclosed early zygote, there was a trend in the direction of increased basal OCR, and FCCP application led to a significant increase in maximal OCR compared to oocytes post-IVM. This pattern suggests an increase in mitochondrial activity and reserve capacity coincident with fertilisation. A peak in mitochondrial activity at fertilisation has previously been shown, first in the sea urchin^35^ and later in the bovine model^36^. It has been proposed that this phenomenon occurs in response to calcium oscillations^37^ which are triggered by PLCζ at the time of fertilisation, and which promote oxygen consumption in oocytes^38,39^. This rise in mitochondrial activity may be due to the ATP demands of chromosome reorganisation for PN formation and extrusion of the second polar body. Demonstration of the presence of a reserve capacity (the difference between maximal and basal OCR) is of note since mitochondrial biogenesis, the normal physiological response to increased ATP demand, does not occur at this stage. However, paternally-derived mitochondria will still be present since they are not degraded until early cleavage^39^ and may contribute to the increased maximal capacity we observed. It is notable that following both IVM and IVF not all oocytes were at the expected developmental stage (Supplementary Figs S4 and S7). Since only 60–70% were expected to be fertilised, this strengthens our observation of an increase in mitochondrial activity at PN-stage.

### Application of extracellular flux analysis to investigate bovine embryo physiology

The OCR did not differ throughout the cleavage stages of bovine preimplantation embryo development. Such a pattern has been previously described for mouse^10^, cow^11,13^, and pig^12^. Upon reaching the blastocyst stage, there was a significant rise in OCR (Fig. 6) - in agreement with earlier reports^9–13^.

The components of OCR were measured in 2-cell embryos and blastocysts. Not unexpectedly, blastocysts exhibited a higher maximal respiratory rate compared to cleavage-stage embryos. This increase in both basal and maximal OCR in blastocysts supports the notion of metabolic plasticity in the preimplantation embryo and provides the facility by which blastocysts can rapidly increase ATP synthesis. ATP demand increases at this stage to meet the needs for Na^+^/K^+^ ATPase activity, necessary for production and maintenance of the blastocoel cavity, to support a rise in protein synthesis, and allow for cellular differentiation^40^. Replication of mtDNA is initiated at the blastocyst stage in the horse^6^.

At the 2–4 cell stage of development, a high degree of heterogeneity in basal OCR and FCCP response was apparent and curiously, a significant proportion of cleavage stage embryos failed to respond to FCCP. In terms of the basal respiration, those cleavage-stage embryos which did not respond to FCCP had a significantly higher basal respiration. Thus, at the 2-cell stage, it was apparent that embryos fell into two distinct groups – one with low OCR and a spare capacity, and one with high OCR and no spare capacity. Nevertheless, the proportion of OCR ascribed to ATP synthesis and non-mitochondrial functions did not differ between the two groups. The reasons behind there being two distinct groups are not immediately clear. However, it has been proposed that the most viable embryos will be metabolically mid-range when distributions of individual values are plotted, while those that exhibit higher or lower metabolic activity are more likely to be stressed^25,41^. Arrest at the 2–4 cell stage is a common phenomenon, representing around embryos 10–15% in human ART^42^ and 15% in the bovine model^43^. A number of factors have been implicated in this developmental arrest including its coinciding with the pressures of embryonic genome activation^44^, chromosomal abnormalities^45^, abnormal cleavage events^46^ and reactive oxygen species (ROS) generation and oxidative stress^47,48^. A preliminary investigation into whether these different groups reflected distinct developmental capacity showed that those in the ‘low’ OCR group were more likely to go on to form blastocysts, suggesting a physiological difference between the groups rather than an observation of timing of cleavage divisions. However, this experiment used ovaries collected on a single day and further work is required to elucidate this intriguing relationship and the mechanism(s) behind it. The use of 6 embryos grouped at random represents a limitation to this finding. The distinct groups observed implies either that the proportion of ‘low’ and ‘high’ OCR embryos affected the resultant reading, or that there might be a paracrine effect from particularly stressed over-active embryos. Our observation could also stem from the stochastic nature of the measurement – it has been demonstrated that each cytokinetic event of cleavage is associated with small peaks in mitochondrial activity^49^.

It is notable that the present values of basal OCR (Fig. 8a) are within the range reported previously, though direct comparisons are difficult due to the differences in technical approach and reporting data. Denuded bovine 1-cell zygotes have been reported to consume approximately 1.78 pmol/min^10^, an increase compared to un-fertilised oocytes. Sugimura *et al*.^34^ reported figures of 0.26 and 0.18 pmol/min prior to and following IVM in denuded bovine oocytes while Lopes *et al*.^36^ found a value of 2.83 pmol/min in mature bovine oocytes and similar values in D3 embryos, with blastocysts undergoing a two to three-fold increase in respiratory activity, though this was affected by morphological quality^21^. Obeidat *et al*.^22^ recently reported denuded bovine oocyte respiration to be approximately 1.2 pmol/min and embryo to be 4.2 pmol/min, though stages were not clearly indicated. These previous studies have therefore reached similar conclusions to our own both in terms of raw numbers but also physiological trends such as a modest increase following fertilisation, and a major increase between cleavage and blastocyst stages.

### Markers of mitochondrial activity across pre-implantation development

Basal and maximal OCR of embryos were significantly below those of cumulus enclosed oocytes at all stages analysed. This most likely reflects the interaction with cumulus that occurs between these stages, since the OCR of denuded oocytes was similar to that measured in early cleavage- stage (2–4 cell) embryos. The contributions of non-mitochondrial OCR, proton leak and coupled respiration expressed as proportions did not differ at any stage of preimplantation development indicating that while overall oxygen consumption might be changing, the components of respiratory function remained similar. We consider this a key finding since it indicates that while alterations in overall respiratory activity occur over the course of development in response to physiological demand, the overall efficiency of the process is unchanged and that the rise in total oxygen consumption is a function of the change in mitochondrial number/mass, substrate availability or rate of activity of the ETC. Around the blastocyst stage, mitochondrial biogenesis begins^6^, consistent with the increase in both basal and maximal OCR observed here. Further, reliance on different fuels (carbohydrates, fats or proteins) can alter mitochondrial activity by affecting the respiratory quotient; i.e. the ratio of CO_2_ production to O_2_ consumption^50^ – thus OCR may be a useful means for non-invasive mapping of changes in nutrient metabolism known to occur across embryogenesis^27^.

Throughout oocyte maturation and preimplantation embryo development, approximately 20% of OCR could be accounted for by proton leak, while about 60% was coupled to ATP formation. Although some minor variations in proportions were observed at the different stages, proton leak and the coupling to ATP production remained relatively constant. The figure of 20% for proton leak has been observed across a range of cell types^51^. Proton leak decreases superoxide production due to its effect on the proton gradient, and is therefore involved in the regulation of ROS production. The process of uncoupling thus has a protective role against ROS, observed for example when under oxidative stress^52^ and in ageing^51^. Coupling efficiency can vary significantly between different cells types, from as low as 30% to up to 90%^14^; differences most likely dependent on ATP demand or in order to regulate ROS levels^52^ and is thought to stem at least in part due to tissue and cell-type specific differences in mitochondrial structure^53^.

Our observation of around 20% of basal OCR derived from non-mitochondrial processes was of particular interest. This figure is higher than for most somatic cells, which tend to give a figure of around 10%^14^, although it was lower than previously determined in rabbit, mouse and bovine oocytes and embryos^34,54,55^ – who reported values of approximately 25%, 23–30%, and 35–40% respectively. This is likely due to biological differences as well as experimental approach. Sources of non-mitochondrial oxygen consumption include cell-surface oxygen consumption from electron transport at the membrane and enzymatic ROS production, for example NADPH oxidase in the rabbit blastocyst^54,56,57^. Cell-surface oxygen consumption has been reported to support rapidly proliferating tumour cells highly active in glycolysis^56^, and might play a similar role in dividing embryos which exhibit aerobic glycolysis^58^.

Spare respiratory capacity was the function with the greatest variation. Overall, a large spare capacity was observed at the stages assessed, though the exception to this generalisation was the sub-population of embryos at the 2-cell stage that did not exhibit spare capacity. The spare capacity may be regulated by the contribution of cumulus cell mitochondria, changes in mitochondrial mass after fertilisation and at the blastocyst stage^6^, and response to physiological demand. This could in part facilitate the peak of increased mitochondrial activity that occurs for example at fertilisation and with cleavage divisions^49^. Reserve capacity has also been demonstrated to be linked to increased cellular survival in fibroblasts and cardiomyocytes^59,60^, ensuring cells have the capacity to provide more ATP should conditions require it. Reserve capacity is facilitated by regulation of the TCA cycle and of complex II of the ETC, which respond to substrate availability^61^. Metabolic sensors trigger this regulation, thus allowing the cell to meet demands and react to stressed conditions. We speculate that the high levels of spare capacity observed throughout may contribute to the metabolic and developmental plasticity that oocytes and embryos show during the periconceptual stage of development that is highly responsive to environmental conditions^62^. Environmental challenges to the embryo may present in the form of maternal diet or *in vitro* culture conditions – conditions under which metabolic activity is altered^34,63^.

### Prospects for the future application of extracellular flux analysis

Overall, we have shown that EFA can readily be used as a tool to measure oxygen consumption in real-time as a proxy for the function of mitochondria and their components (spare respiratory capacity, proton leak, non-mitochondrial and coupled respiration) in small groups of mammalian oocytes and preimplantation embryos This means that the components of respiratory activity can now be measured in mammalian oocytes and early embryos opening up a new area of investigation into the detailed bioenergetics of these developmental processes. Furthermore, given the simplicity of the approach, we propose that EFA will have great utility in enhancing our understanding of the effects of composition of embryo culture media on metabolic regulation in early embryos. The approach is robust and rapid, and its automated nature limits room for operator-induced error and potential harm to the gametes and embryos. Crucially, analyses are non-invasive and do not impact on ongoing *in vitro* development. Future work may lead to optimisation of the Seahorse XFp system to enable higher sensitivity, allowing it to be applied to single oocytes and embryos, with the potential to screen individual embryos prior to transfer in clinical and farm animal IVF.

## Materials and Methods

### *In vitro* production (IVP) of bovine embryos

IVP was performed as described previously^64^. Unless stated otherwise, all media were prepared in the laboratory. Ovaries were collected from a local abattoir on the day of slaughter and transported in PBS warmed to 39 °C within 3 hours of slaughter. Ovaries were washed twice in warmed PBS, and follicles were aspirated into warmed Hepes-buffered M199 containing heparin. Oocytes with compact cumulus were matured in groups of 50 for 18–22 hrs in 5% CO_2_, 20% O_2_ in 500 μl M199 containing 10% FBS supplemented with FSH, LH, EGF and FGF (BMM) at 39 °C. Fertilisation was performed by co-incubating 0.5 × 10^6^ sperm/ml with groups of 50 oocytes in 500 μl Fertilisation-TALP (Fert-TALP) for 24 hours. Presumptive zygotes were then fully denuded of cumulus cells by vortexing, and transferred into 20 μl drops of synthetic oviduct fluid (SOF) containing BSA and amino acids in groups of 20. These were incubated at 39 °C in 5% O_2_, 5% CO_2_, 90% N_2_.

Oocytes or embryos were selected at the desired stage following standard IVP (described above), with the exception of GV-stage oocytes which were cultured overnight in cycloheximide (10 µg/ml) to synchronize their stage. OCR was measured in groups of 6 CEOs following 24 hour culture in the presence of either meiosis-II (M-II) inhibitor cycloheximide, to prevent GVBD and resumption of meiosis, or of maturation-promoting hormones, to support progression to M-II stage. Expected proportion of oocytes at M-II stage after IVM was assessed by three independent nuclear staining experiments (0.1% Hoechst in 100% ethanol) following 18–22 h IVM (Supplementary Fig. S7). Presumptive zygotes were selected after 9 hours co-incubation with motile sperm, washed to remove sperm and stripped to corona. In order to assess approximate timing for appearance of PN following fertilisation under our experimental conditions, independent staining experiments were performed. Oocytes were stained using 0.1% Hoechst 33342 between 4 and 12 hours following addition of sperm. We determined in independent experiments that 9 hours of co-incubation with motile sperm resulted in the highest concentration of PN-stage zygotes (Supplementary Fig. S4). Embryos were selected out at the appropriate time (2–4 cell D2-D3, 8–16 cell D3-D4, blastocyst D6-D8) based on visual morphological staging.

Oocytes were analysed in BMM, PN-stage zygotes in HEPES-buffered Fert-TALP, and embryos in HEPES-buffered SOF – with all stages being analysed in groups of 6 in 180 μl media. Equine COCs were analysed in groups of 3. Oocytes were fully denuded using 0.1% hyaluronidase and 1 minute vortexing, or stripped to corona using a holding pipette.

### *In vitro* maturation of equine oocytes

Equine COCs were collected from abattoir-derived ovaries and held overnight as previously described^65,66^. IVM was performed for 30 h in groups of three COCs with compact cumulus directly in a Seahorse XFp Bioanalyser plate in 180 µl of maturation media (M199 with Earle’s salts, 10% FBS, 25 µg/ml gentamicin with 5 mU/ml FSH^66^. Only COCs with compact cumulus were used for this experiment.

### Preparation of mouse oocytes

CD-1 (Charles River, Tranent, UK) mice were maintained, superovulated and housed overnight with stud males for mating as previously described by Ruane *et al*.^67^. Denuded fail to fertilise M-II oocytes (n = 8) were obtained 36 h post-ovulation and cultured briefly (2–4 h) in 50 µl KSOM medium (Millipore) containing 0.4% BSA at 37 °C, 5% CO_2_ in air under ovoil (Vitrolife, Göteborg, Sweden) prior to assay in the XFP in a single group of 8.

### Preparation of human oocytes

Appropriate consent to donate to research was obtained from patients undergoing Controlled Ovarian Stimulation (COS) for Intra-cytoplasmic Sperm Injection (ICSI) treatment in a National Health Service (NHS) IVF clinic as previously described^68,69^. Ethics approval was obtained from the Manchester University Hospitals Research Ethics Committee and all donations were in accordance with the Human Fertilisation and Embryology Authority (HFEA) research licence R0026. All oocytes (n = 5) were denuded MII failed to fertilise (OPN) fresh oocytes obtained from a single patient 18–20 h post ICSI. All oocytes were previously cultured in 50ul GTL medium under Ovoil (Vitrolife, Göteborg, Sweden) at 37 °C, 5% O_2_ and 6% CO_2_ prior to assay in the XFP, 20–24 h post ICSI in a single group of 5 oocytes.

### Application of Seahorse XFp to measure oxygen consumption in oocytes and embryos

Sensor-containing Seahorse fluxpaks (Agilent Technology) were incubated overnight at 37 °C in a non-CO_2_ humidified incubator. The minimum time for incubation accepted was 8 hours and maximum 36 hours. The sensor-containing fluxpak was calibrated for approximately 15 minutes as per manufacturer guidance. Upon completion, the pre-warmed cell plate containing biological material was loaded into the machine. Oocytes and embryos were analysed using a specialised protocol involving a 12 minute equilibration period upon loading the cell plate, and alternating between a 3 minute measurement period and a 1 minute re-equilibration period. The measurement period involves the lowering of a sensor-containing probe, which creates an airtight 2.3 µl microenvironment in which change in pressure in mmHg is measured over time. This is followed by a 1 minute period in which the probe is lifted, and the 180 μl well re-equilibrates. Plate specific ‘blank’ cell-free wells containing culture medium are used to account for environmental changes and flux of oxygen in the absence of cells, and as such OCR is given as a function of these blank cell-free wells, with the flux in oxygen accounting for plasticware, diffusion of atmospheric O_2_, and finally specimen consumption. Output of Seahorse was given as OCR in pmol/min/well.

To confirm that the assay had no impact on ongoing development, the blastocyst rates of bovine embryos which had undergone basal EFA on D2 were compared to those which were moved into HEPES SOF and kept in non-gassed incubation for the entirety of the Seahorse analysis (approximately 1 hour) as a control. Blastocyst rate was assessed daily between D6 and D8. Blastocyst rates observed in both groups were similar to expected rates given our IVP protocols (data not shown).

Both mouse and human embryos were analysed in the same manner – using 8 oocytes and 5 oocytes per well respectively. For equine COCs, the procedure was as described above with the following exception. As IVM took place directly in the cell plate (3 COCs per well), it was removed from the incubator and placed in the analyser at two different time points (4 and 28 hours after the start of IVM; IVM + 4 hours, and IVM + 28 hours). After each set of readings was complete the plate was placed back in the incubator.

### Use of mitochondrial inhibitors

Mitochondrial inhibitors were dissolved in 100% ethanol at 1000x the working stock. These were stored at −20 °C for up to 3 months. Inhibitors were diluted in warmed analysis media (BMM, TALP or SOF, as dependant on stage of analysis) within 30 minutes of starting the assay.

Optimisation was carried out on GV-stage oocytes to establish appropriate concentrations of mitochondrial inhibitors indicative of mitochondrial parameters (Supplementary Fig. S3). This involved serial injections of (1) oligomycin, (2) FCCP, and (3) a combination of antimycin A and rotenone. The Seahorse-XFp (Agilent) recommended concentration of 1 µM oligomycin was used as a starting point, as it has been previously established to be appropriate for most cell types (Agilent Seahorse XF Cell Mito Stress Test Kit User Guide, 2017). This was tested in addition to 1.25, 2 and 3 μM. FCCP was tested at a variety of concentrations, using a titration of low (0.25 to 2 µM) and high ranges (2.5 to 7.5 µM). Antimycin A/Rotenone were tested at three concentrations: 1.5, 2.5 and 3.75. The lowest effective concentration was selected for each drug. Drug concentrations established for oocytes were applied to embryos. Oligo and A/R concentrations were effective on embryos, however FCCP was deemed too high, reducing OCR compared to basal, and was reduced to 3.75 µM (Supplementary Fig. S5).

Inhibitors were loaded into the cell plate such that each injection represents 10% of the total well volume: 20 µl, 22 µl, 25 µl and 27 µl. As such, working concentrations were 10x the desired well concentration.

### Data interpretation and statistical analysis

Wave software (Agilent Technologies) was used to determine oxygen consumption in pmol/min/well. This was normalised to number of oocytes/embryos per well. The third basal point, deemed most stable, was used as point of comparison for all data presented as proportion of basal. Point of highest response was used for all analysis for mitochondrial inhibitors. GraphPad Prism was used for all statistical analysis, using a significance level of p < 0.05. Unpaired t-test was used where two groups were being compared. One-way ANOVA was used where basal OCR in more than two groups were being compared, while two-way ANOVA was used when drug response was considered between stages. Number of wells are used as technical replicates. Arc-sine transformation was used for the analysis of all proportional data. Number of wells in addition to number of oocytes/embryos used at each stage is described in Table 1. All graphs are presented as mean ± SEM.

**Table 1:** Experimental design.

Figure	Stage	Investigation	Number of experimental replicates	Number of wells analysed	Number of biological replicates
1	GV-stage	Variable cumulus contribution	3	6/group	36/group
2	M-II stage	Application to mouse and human oocytes	1	1/mouse 1/human	8 mouse 5 human
3	GV-stage	Mitochondrial inhibitor treatment	3	12 treated 13 untreated	72 78
4	GV-stage M-II stage PN stage	Impact of IVM and IVF	3	12 GV 11 M-II 13 PN	72 66 78
5	IVM +4 IVM +28	Impact of IVM (equine)	3	9	27
6b	2–4 cell 8–16 cell Blastocyst	Impact of embryonic development	3	11 2–4 cell 13 8–16 cell 16 blastocysts	66 78 96
6c	Blastocyst	Blastocyst stage	3	8/stage	48/stage
6d	2–4 cell Blastocyst	Mitochondrial parameters in the embryo	3	11/group	66/stage
7	2–4 cell	Spare capacity	3	12	72

Experimental design for Figs 1–5 is described, defining the variable of investigation, the developmental stage used and the number of samples per group. The ‘number of experimental replicates’ refers to pooled oocytes/embryos from the same ovary collection, ‘number of wells’ the number of Seahorse XFp wells analysed, and ‘number of biological replicates’ the number of oocytes/embryos used overall. For example, in Figs 1, 6 wells of 6 oocytes are analysed per group, making 36 the total number of biological replicates; in Fig. 2 a single well was analysed for each species containing 8 mouse and 5 human oocytes respectively.

### Ethics and consent

All work on human oocytes was carried out with full informed consent and full ethical approval.

## Supplementary information

Supplementary data

## Data Availability

The authors confirm that all source data will be deposited on an institutional data repository and made available upon request to the senior author.
