# Supplementary material for: Application of extracellular flux analysis for determining mitochondrial function in mammalian oocytes and early embryos
Source: Sci Rep. 2019 Nov 14;9:16778. doi: 10.1038/s41598-019-53066-9 (PMC6856134; doi:10.1038/s41598-019-53066-9)
Supplement: Supplementary file 1 — Supplementary data [file 41598_2019_53066_MOESM1_ESM.pdf]

Application of extracellular flux analysis for determining mitochondrial function in mammalian oocytes and early embryos

Bethany Muller[1], Niamh Lewis[2] , Tope Adeniyi[3], Henry J Leese[1], Daniel R Brison[3,4], Roger G Sturmey[1]

[1] Centre for Atherothrombosis and Metabolic Disease, Hull York Medical School, University of Hull, Hull, HU6 7RX, UK

[2] Institute of Aging and Chronic Disease, University of Liverpool, UK

[3] Department of Reproductive Medicine, Manchester University NHS Foundation Trust, Manchester Academic Health Sciences Centre UK

[4] Maternal and Fetal Health Research Centre, School of Medical Sciences, University of Manchester UK

Supplementary data

Figure S1

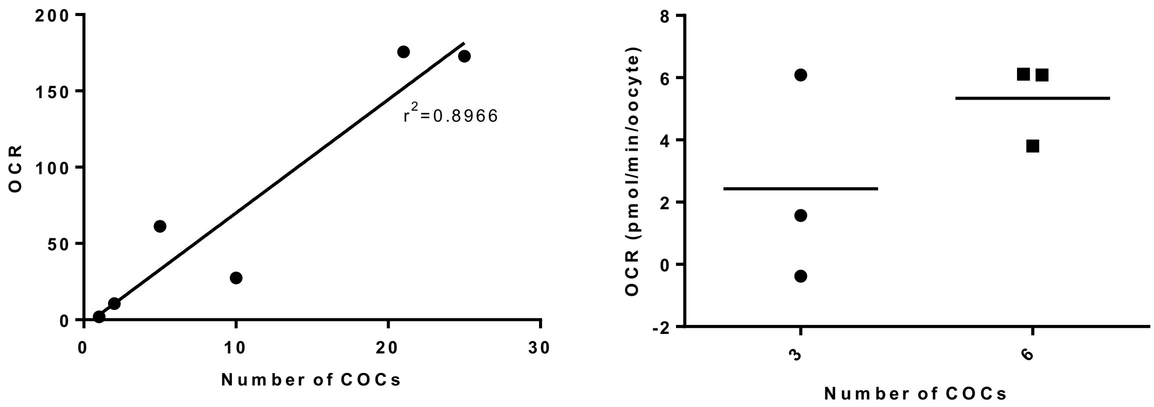

Figure S1│ Determination of group size for oxygen consumption measurements in bovine COCs. (a) The linearity of OCR reading for between 1 and 25 oocytes (n=1) which shows significant correlation between number of oocytes per well and measured value of OCR (p=0.0042). (b) A comparison between basal OCR for Seahorse XFp analysis of groups of 3 or 6 COCs (n=1).

Figure S2

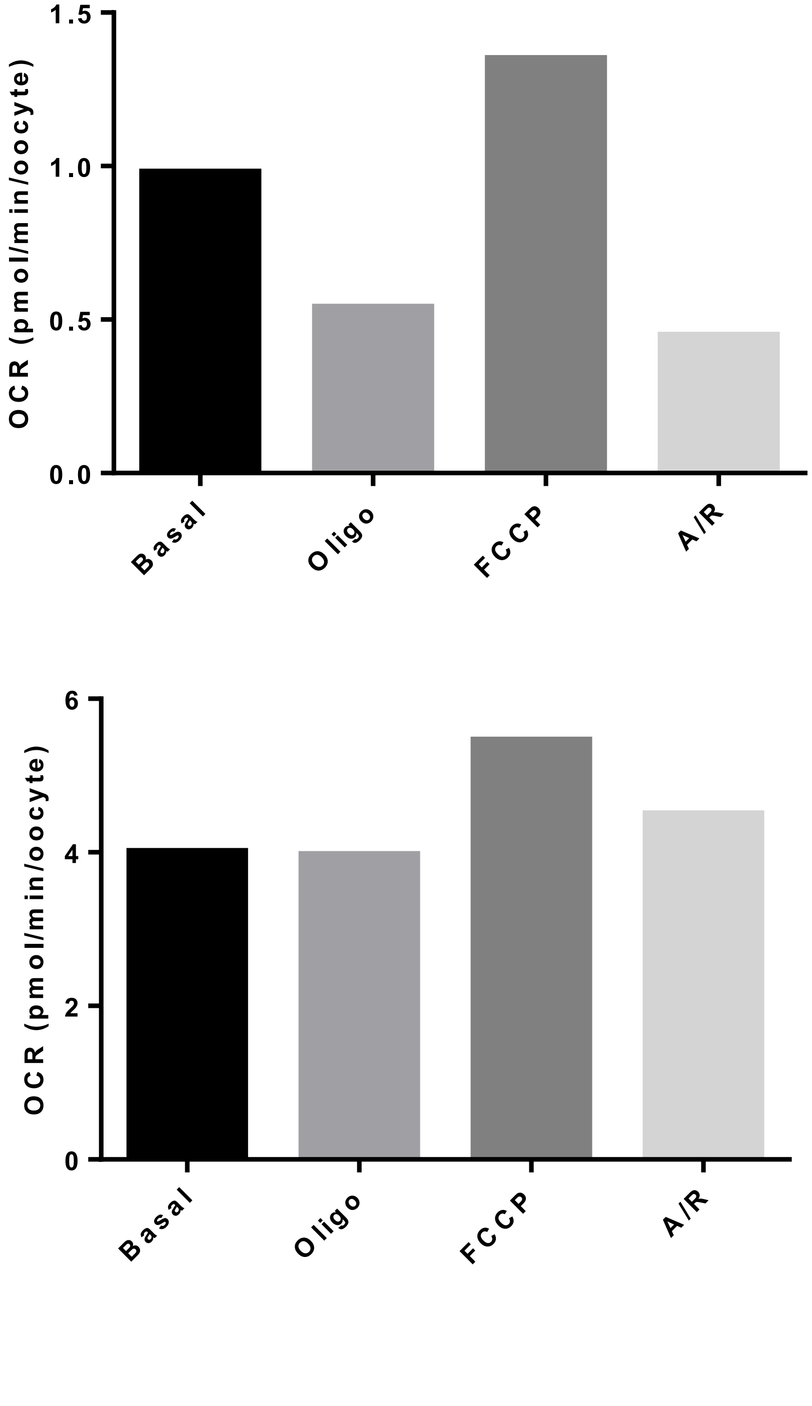

Figure S2│Application of mitochondrial inhibitors to denuded mouse and human M-II oocytes. Mitochondrial inhibitors oligomycin (1 µM), FCCP (5 µM) and antimycin A and rotenone (2.5 µM) were applied in sequence in (a) Mouse oocytes (n=1, representative of 8 oocytes), and (b) Failed-to-fertilise human oocytes (n=1, representative of 5 oocytes). Each data set represents a single data point.

Figure S3

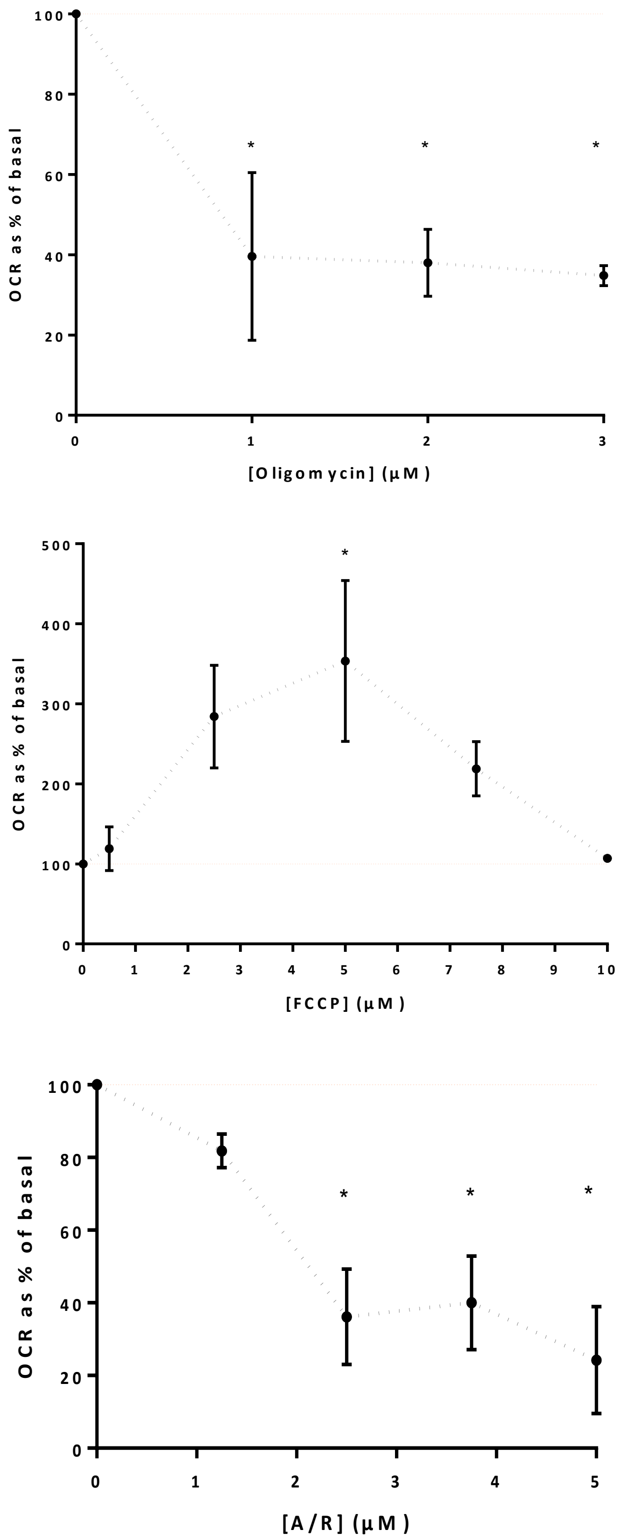

Figure S3│Optimisation of drug concentrations used for dissecting the components of oxygen consumption in bovine oocytes. (a-c) Rate vs. dose plots for inhibitors. Final concentrations chosen were verified in at least two independent experiments. Data shows mean ± SEM. Significance assessed using one-way ANOVA, to $p<0.05$. Each point is representative of a minimum of two experimental replicates (12 CEOs).

Figure S4

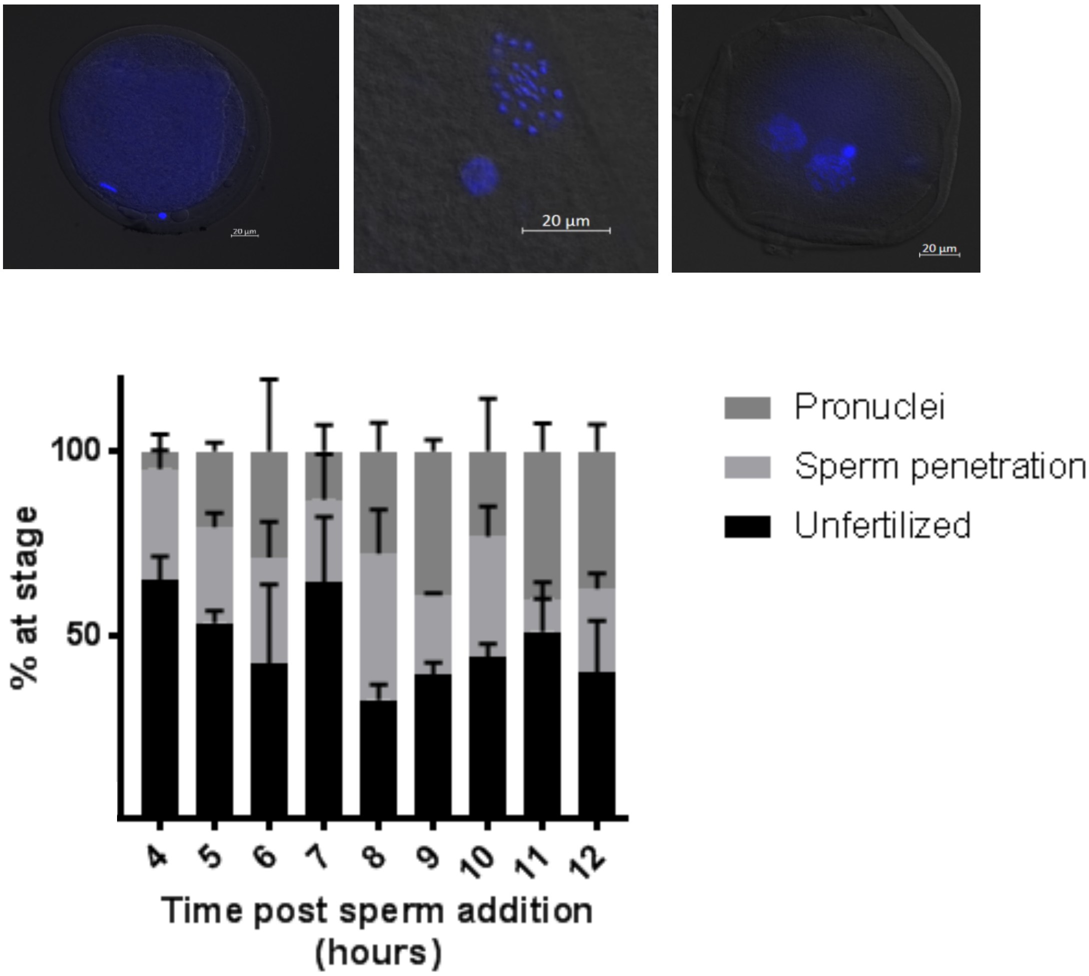

Figure S4 │ Determination of timing of PN stage in bovine embryos. Hoechst staining was used to determine timing for pronuclear formation in bovine embryos following IVP procedures at 4-12 hours following the addition of motile sperm. (a) Representative images showing Hoechst nuclear staining in bovine zygotes, indicating (i) unfertilized oocyte, (ii) sperm penetration, and (iii) presence of two pronuclei. (b) The breakdown of zygote stage at 4-12 hours post sperm addition. N=3, mean ± SEM, 10 oocytes assessed at each time point per experiment.

Figure S5

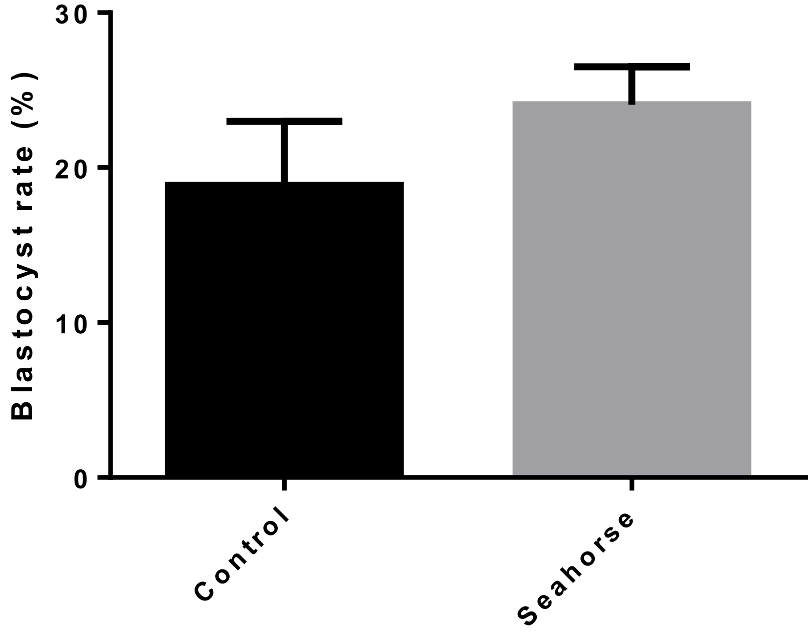

Figure S5 │ Blastocyst rate following 1 hour EFA on D2. Embryos were either subjected to a 1 hour basal OCR measurement (12 measurements) (Seahorse) or moved into HEPES SOF for the time of the assay (control) before being combined into groups of 15-18 embryos for standard embryo culture. Blastocyst rate was assessed daily from D6 to D8. N=3, representing 6 culture drops per group.

Figure S6

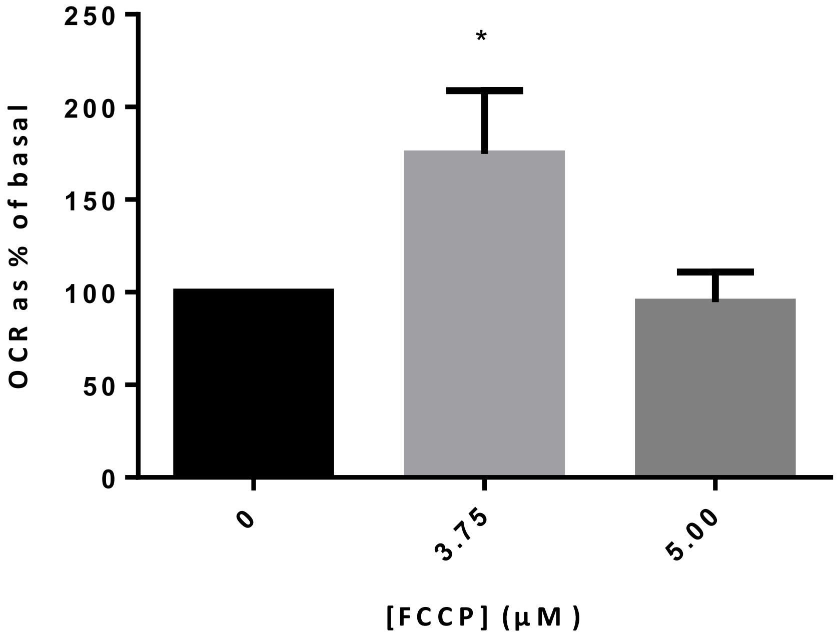

Figure S6 │ FCCP drug optimization in bovine embryos. FCCP drug optimization at the 2-4 cell cleavage stage. Mean ± SEM (n=3, representative of 18 embryos for each concentration). 100% represents basal, and drug response is shown as a proportion of this. Oligomycin and Antimycin/Rotenone produced the expected responses and thus were used at 1 and 2.5µM respectively. * indicates $p<0.05$.

Figure S7

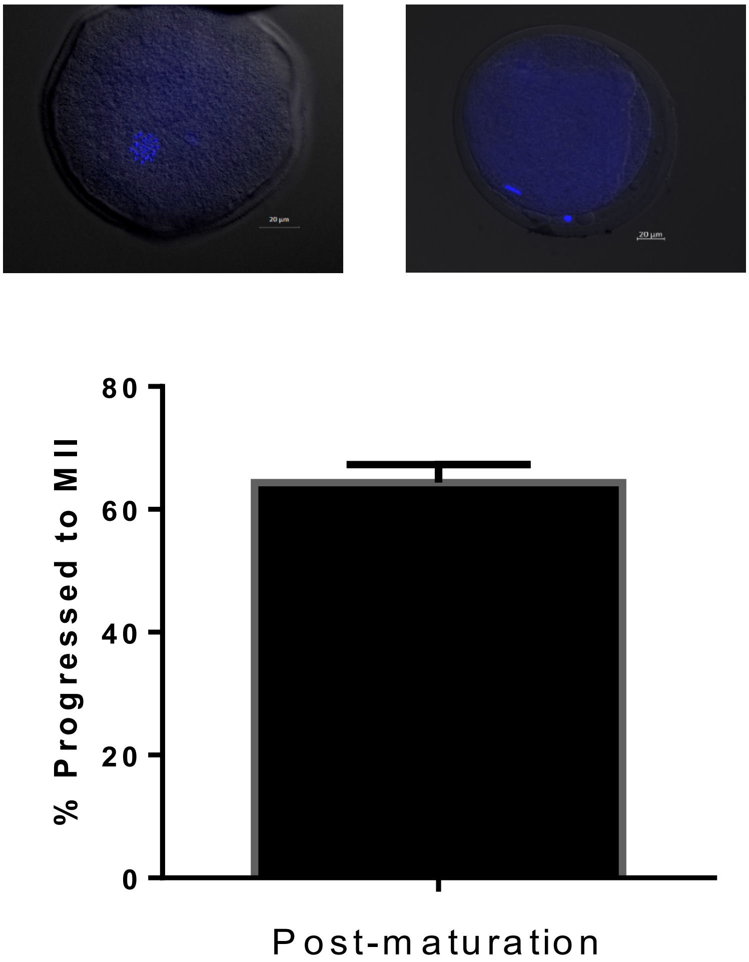

Figure S7 │ Nuclear status following IVM protocol in bovine COCs. Hoechst staining was applied to determine nuclear status of oocytes following 18-22hr culture in BMM. (a) Representative images showing oocytes at (i) GV and (ii) M-II stage. (b) Percentage of oocytes at M-II stage following IVM. Data represents mean ± SEM (n=3, representing 10 oocytes per experiment).
